# Supplementary material for: Location-dependent maintenance of intrinsic susceptibility to mTORC1-driven tumorigenesis
Source: Life Sci Alliance. 2019 Mar 25;2(2):e201800218. doi: 10.26508/lsa.201800218 (PMC6435042; doi:10.26508/lsa.201800218)
Supplement: Supplementary file 1 [file LSA-2018-00218_TableS1.doc]

**Table S1. Antibody Clones and Dilutions**

| Antibody | Vendor | Catalog # |
| --- | --- | --- |
| Rabbit anti-Phospho-S6 Ser240/244 conjugated to Ax488 (clone D68F8) diluted 1:400 | Cell Signaling Technology | Cat# 5018S |
| Rabbit anti-Phospho-S6 Ser240/244 conjugated to PE (clone D68F8) diluted 1:200 | Cell Signaling Technology | Cat#14236S |
| Rabbit anti-Phospho-S6 Ser240/244 conjugated to Ax647 (clone D68F8) diluted 1:800 | Cell Signaling Technology | Cat#5044 |
| Rabbit anti-Phospho-4E-BP1 Thr37/46 conjugated to PE (clone 236B4) diluted 1:100 | Cell Signaling Technology | Cat#7547S |
| Rabbit anti-Phospho-4E-BP1 Thr37/46 conjugated to Ax647 (clone 236B4) diluted 1:100 | Cell Signaling Technology | Cat# 5123S |
| Mouse anti-Human Stat3, Phospho Ser727 conjugated to PE (clone 49/p-Stat3) diluted 1:15 | BD Biosciences | Cat# 558557 |
| Rabbit anti-Phospho-S6 Ser235/236 conjugated to PE (clone D57.2.2E) diluted 1:100 | Cell Signaling Technology | Cat#4858 |
| Rabbit anti-Phospho-p44/42 MAPK (Erk1/2) Thr202/Tyr204 Conjugated to PE (197G2) diluted 1:50 | Cell Signaling Technology | Cat# 14095S |
| Rabbit anti-Phospho-p44/42 MAPK (Erk1/2) Thr202/Tyr204 Conjugated to Ax647 (197G2) diluted 1:50 | Cell Signaling Technology | Cat#13148S |
| Rat anti-Mouse CD133 (Prominin) Conjugated to Ax488 (clone 13A4) diluted 1:300 | eBioscience (Thermo Fisher) | Cat#53-1331-80 |
| Mouse anti-GFAP conjugated to BUV395 (clone 1B4) diluted 1:200 | BD Pharmigen | Custom Conjugate |
| Mouse anti-GFAP conjugated to Ax647 (clone 1B4) diluted 1:200 | BD Pharmigen | Cat#561470 |
| Rabbit anti-Doublecortin (DCX) conjugated to Pacific Blue* (clone 4604S) *in-house conjugation diluted 1:60 | Cell Signaling Technology | Cat#4604BF |
| Mouse anti-Doublecortin (DCX) conjugated to PE (clone 30/doublecortin) diluted 1:60 | BD Pharmigen | Cat#561505 |
| Rat anti-Mouse CD24 conjugated to BUV395 (clone M1/69) diluted 1:800 | BD Pharmigen | Cat#744471 |
| Rat anti-Mouse CD24 conjugated to BUV395 (clone M1/69) diluted 1:800 | BD Pharmigen | Cat#562563 |
| Rat anti-Mouse EGFR conjugated to biotin (clone BAF 1280) diluted 1:200 | R&D Systems | Cat#1280-ER |
| Streptavidin, conjugated to BV786 diluted 1:200 | BD Horizon | Cat#563858 |
| Mouse anti-p38 MAPK phospho Thr180/Tyr182 conjugated to Pacific Blue (clone 36/p38) diluted 1:50 | BD Biosciences | Cat#560313 |
| Mouse anti-PLCγ phosphoTyr759 conjugated to PE (clone K86-689.37) diluted 1:50 | BD Phophoflow | Cat#558490 |
| Mouse anti-Human Akt phospho Ser473 conjugated to Ax488 (clone M89-61) diluted 1:50 | BD Pharmigen | Cat#560404 |
| Mouse anti-S6 Ribosomal Protein conjugated to PE (clone 54D2) diluted 1:50 | Cell Signaling Technology | Cat#55594 |
| Rabbit anti phospho-S6 Ser240/244 (clone D68F8) diluted 1:800 | Cell Signaling Technology | Cat#5364 |
| Rabbit anti phospho-S6 Ser235/236 (clone D57.2.2E) diluted 1:800 | Cell Signaling Technology | Cat#4858 |
| Rabbit anti phospho-4E-BP1 Thr37/46 (clone 236B4) diluted 1:800 | Cell Signaling Technology | Cat#2855 |
| Chicken anti-GFAP Polycolonal antibody (clone Ab4674) 1:2500 | Abcam | Cat#Ab4674 |
| Guinea Pig anti-DCX (clone AB2253) diluted 1:1000 | Millipore | Cat#AB2253 |
| Mouse anti-Mash1 (clone 24B72D11.1) diluted 1:200 | BD Pharmigen | Cat#556604 |
| Rabbit anti-Nkx2.1/TTF-1 (clone D2E8) diluted 1:50 | Cell Signaling Technology | Cat#12373 |
| Rabbit anti-Nkx2.1/TTF-1 diluted 1:100 | Santa Cruz | Cat#sc-13040 |
| Rat anti-RFP (clone 5F8) diluted 1:1000 | Allele Biotech | Cat# ACT-CM-MRRFP10 |
| Chicken anti-Vimentin (clone AB5733) diluted 1:1000 | Millipore | Cat#AB5733 |
| Mouse anti-β-catenin (clone 14/Beta-Catenin) diluted 1:1000 | BD | Cat# 610153 |
| Mouse anti-γ-tubulin (clone GTU-88) diluted 1:500 | Abcam | Cat#AB11316 |
| Rabbit anti-Emx1 (clone PA5-35373) diluted 1:50 | Thermo Fisher | Cat#PA5-35373 |
| Rabbit anti Pax6 (clone Poly19013) diluted 1:500 | Biolegend | Cat#901310 |
